# The Impact of Hepatitis B Vaccination Status on the Risk of Diabetes, Implicating Diabetes Risk Reduction by Successful Vaccination

**DOI:** 10.1371/journal.pone.0139730

**Published:** 2015-10-28

**Authors:** Jean Huang, Horng-Yih Ou, James Lin, Rudruidee Karnchanasorn, Wei Feng, Raynald Samoa, Lee-Ming Chuang, Ken C. Chiu

**Affiliations:** 1 Department of Clinical Diabetes, Endocrinology, and Metabolism, City of Hope National Medical Center, Duarte, California, United States of America; Division of Endocrinology, Metabolism and Nutrition, Department of Internal Medicine, Harbor-UCLA Medical Center, Torrance, California, United States of America; 2 Division of Endocrinology and Metabolism, Department of Internal Medicine, National Cheng-Kung University Medical College and Hospital, Tainan, Taiwan; 3 Department of Gastroenterology, City of Hope National Medical Center, Duarte, California, United States of America; 4 Division of Endocrinology, Department of Medicine, University of Kansas Medical Center, Kansas City, Kansas, United States of America; 5 Department of Internal Medicine, National Taiwan University Hospital, Taipei, Taiwan; Graduate Institute of Preventive Medicine, School of Public Health, National Taiwan University, Taipei, Taiwan; Centers for Disease Control and Prevention, UNITED STATES

## Abstract

**Background:**

The liver plays a key role in fuel metabolism. It is well established that liver disease is associated with an increased risk for diabetes mellitus. Hepatitis C virus infection has been known to increase the risk of diabetes. However, much less is known about the role of hepatitis B virus (HBV) infection in diabetes. We examined the association of diabetes based on the vaccination status for HBV.

**Methods:**

In this cross-sectional study, we included adult subjects (≥20 y/o) with HBV serology available from the National Health and Nutrition Examination Survey 2005–2010. Diabetes was defined as established diabetes or fasting plasma glucose concentration ≥7.0 mmol/L, 2-hour plasma glucose concentration ≥11.1 mmol/L, or HbA1c ≥ 47.5 mmol/mol (6.5%). Vaccination was based on the reported history and immunization was determined by HBV serology. The odds ratio (OR) with 95% confidence intervals (95% CI) were calculated with consideration of the following covariates: age, gender, BMI, ethnic/racial group, current smoker, current alcohol consumption, family history of diabetes, poverty index, and education.

**Results:**

This study included 15,316 subjects. Among them, 2,320 subjects was immunized based the HBV serology. Among 4,063 subjects who received HBV vaccination, successful vaccination was only noted in 39% of subjects. The HBV vaccination was not associated with diabetes (OR: 1.08, 95%CI: 0.96–1.23). Serology evidence of HBV immunization was associated with a reduced OR of diabetes (0.75, 95%CI: 0.62–0.90). Successful HBV vaccination was also associated with a reduced OR of diabetes (0.67, 95%CI: 0.52–0.84).

**Conclusions:**

Although our study shows the association of HBV vaccination with the reduced odds of diabetes by 33%, a prospective study is warranted to confirm and examine the impact of HBV vaccination in prevention of diabetes.

## Introduction

Diabetes affects 9.3% of the US population with 21 million diagnosed and 8.1 million undiagnosed [1]. The prevalence of diabetes has increased substantially [2] and consequently so has its financial impact on society with the estimated cost of 245 billion in the US [1]. It is projected that by 2030, the global burden of diabetes in 2030 is 366 million (about 4.4% of the world’s population) with the greatest impact on developing countries and about 50% of diabetes in Asian and Pacific regions [3]. As diabetes is a highly prevalent disease with a significant and potentially life-threatening co-morbidity and financial burden, the identification of causative factors could potentially lead to better treatment and prevention strategies.

Diabetes is a syndrome complex with a common manifestation of hyperglycemia of diverse genetic and non-genetic etiologies. Infection has been implied to play a role in the pathogenesis of diabetes. Among them, the role of hepatitis C virus infection in diabetes is well recognized [4–6]. In contrast, there is much less information available about the role of hepatitis B virus (HBV) in diabetes. The rate of new HBV infections has declined significantly by about 75% in the United States since 1991, when a HBV vaccination program was implemented [7]. Despite this success, HBV infection remains a significant public health issue with up to 1.4 million carriers in the United States [8]. Furthermore, HBV infection is a major global health threat with about 350 million chronic HBV infection worldwide, especially in Asian and Pacific regions [8] where the prevalence of diabetes is recently noted to increase drastically [3].

The role that HBV plays in diabetes is less clear. Although the association with gestation diabetes has been reported [9], chronic HBV infection is not found to increase the risk of diabetes [10]. Another study demonstrated the association of HBV infection with diabetes in Asian American, but not in Pacific Islanders [11]. To further elucidate the relationship of HBV and diabetes, this study examined the data derived from the National Health and Nutrition Examination Survey (NHANES) 2005–2010. To our knowledge, this is the very first study examining the role of HBV vaccination and immunization in diabetes in large sample with multiple ethnic/racial groups. Although diabetes is also preventable with dietary change, physical activity, behavior modification, and various pharmacological interventions, the results of the present study may provide a new method of prevention via vaccination.

## Materials and Methods

### Ethnics statement

The National Health and Nutrition Examination Survey (NHANES) is a program of studies designed to assess the health and nutritional status of adults and children in the United States. The survey is unique in that it combines interviews and physical examinations. NHANES is a major program of the National Center for Health Statistics (NCHS). NCHS is part of the Centers for Disease Control and Prevention (CDC) and has the responsibility for producing vital and health statistics for the Nation. In 1999, the survey became a continuous program that has a changing focus on a variety of health and nutrition measurements to meet emerging needs. Data collection for (NHANES 2005–2010) was approved by the NCHS Research Ethics Review Board (http://www.cdc.gov/nchs/nhanes/irba98.htm). Informed consent was obtained from participants. The records/information was anonymized and de-identified prior to release in the NHANES website (http://www.cdc.gov/nchs/nhanes/about_nhanes.htm). Analysis of de-identified data from the survey is exempt from the federal regulations for the protection of human research participants. Only de-identified data from the survey was used in this study.

### Studied subjects

**Fig 1:**
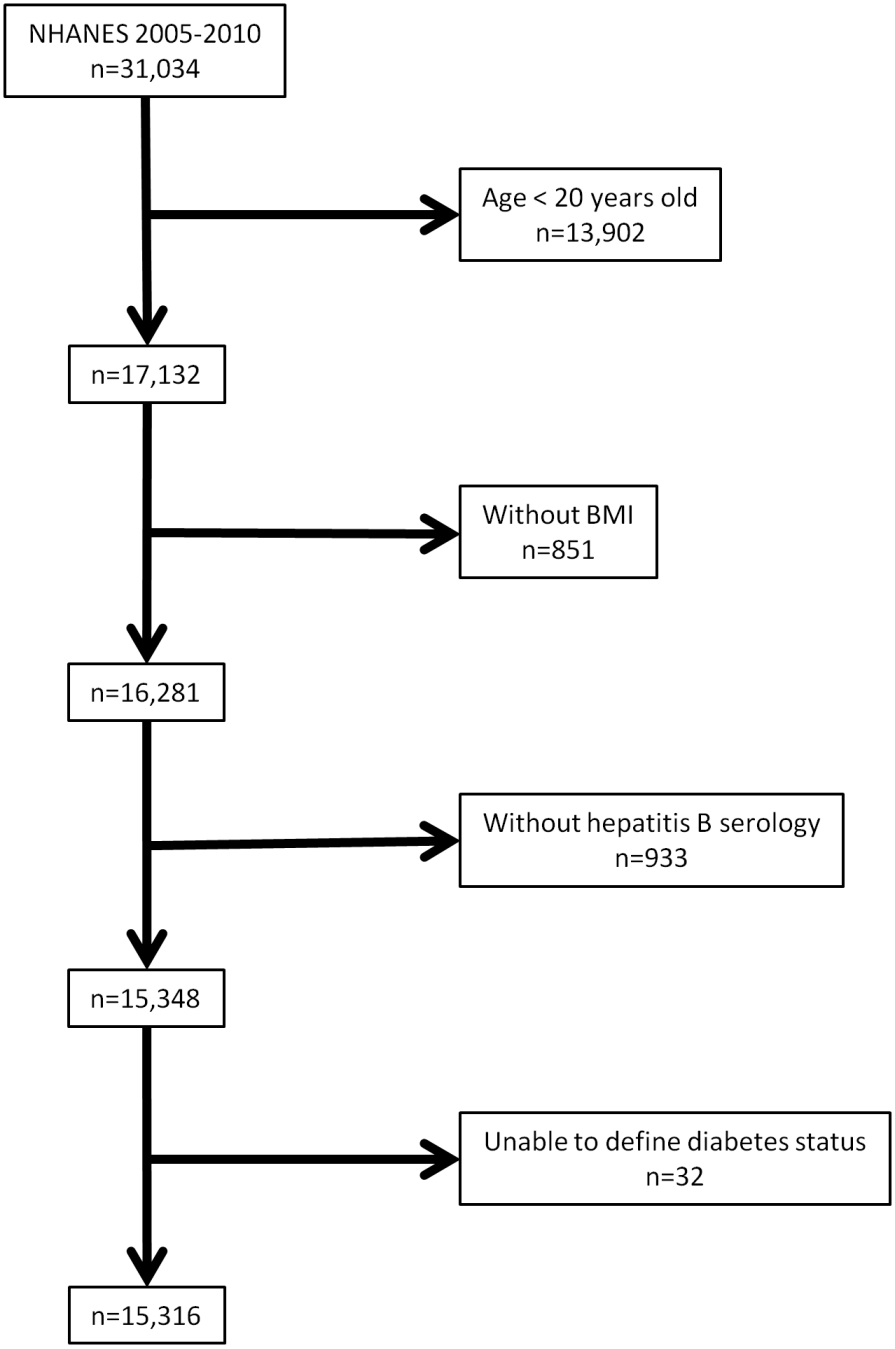
Sampling scheme.

Data from the NHANES from 2005 through 2010 were evaluated for the study (n = 31,034). As shown in Fig 1, we excluded 13,902 subjects who were younger than 20 years of age at the time of the screening interview and 851 subjects without body mass index (BMI) information. Then, we excluded 933 subjects whom hepatitis B serology was not available and 32 subjects whose diabetes status could not be determined by either history or laboratory measurement as described below.

### Definition of diabetes

Established diabetes (n = 1,807) was based on the history of self-reported diabetes or by the use of insulin and/or oral anti-diabetic agents. Diabetes was defined in accordance with the guidelines set forth by the American Diabetes Association [12]. As treatment affected HbA1c, fasting plasma glucose concentration and 2-hour plasma glucose concentration, 658 subjects with established diabetes were noted to have HbA1c <47.5 mmol/mol, 330 subjects with established diabetes were noted to have fasting plasma glucose concentration <7.0 mmol/L, and 31 subjects with established diabetes were noted to have 2-hour plasma glucose concentration <11.1 mmol/L. Diabetes was defined as established diabetes or fasting plasma glucose concentration ≥7.0 mmol/L, 2-hour plasma glucose concentration ≥11.1 mmol/L, or HbA1c ≥47.5 mmol/mol (6.5%). In this sample set, we were able to define diabetes in 2,626 subjects by above criteria. Since sampling weights were only available for subsampling for plasma fasting glucose and 2-hour plasma glucose concentrations, multiple criteria, in addition to plasma fasting glucose and 2-hour plasma glucose concentrations, were used defined diabetes in the present study. Thus, sampling weights were not used in this study.

### Hepatitis B vaccination history

The information on the hepatitis B vaccination was categorized as receiving all 3 doses or less than 3 doses. Among them, 91.07% of vaccinated subjects received all 3 doses of hepatitis B vaccination. As the analysis was mainly based on the serological results, we did not separate the subjects received all 3 doses or less than 3 doses.

### Laboratory methods

#### Plasma glucose

Fasting plasma glucose concentrations were obtained from subjects examined in the morning after a 9 hour fast. After fasting samples were obtained, subjects were asked to drink a calibrated dose (75 grams) of glucose with a second venipuncture 2 hours (± 15 minutes) later for 2-hour plasma glucose concentrations. Plasma glucose concentrations were determined by a hexokinase method. Due to different laboratory instruments were used in 2005–2006 and 2007–2010, we applied regression equations to align fasting plasma glucose and 2-hour plasma glucose concentrations obtained in 2005–2006 as recommended by the NHANES.

#### HbA1c

HbA1c was measured using high performance liquid chromatography based assays. Although different HbA1c laboratory instruments and laboratories were used between 2005 and 2010, both laboratories were standardized by participating in the National Glycohemoglobin Standardization Program (NGSP). The NGSP reviewed and concluded that both NHANES laboratories met NGSP criteria for bias and precision from 1999–2010. As recommended by the NGSP, no cross-over regression was made in the present study. The re-released HbA1c data for 2007–2008 (GHB_E) and 2009–2010 (GHB_F) in March 2012 were used in this study.

#### Hepatitis B surface antibody (HBsAb)

The AUSAB Enzyme immunoassay for HBsAb used the “sandwich principle” a solid phase enzyme-linked immunoassay technique to detect HBsAb levels in serum or plasma.

#### Hepatitis B core antibody (HBcAb)

The Ortho HBc ELISA Test System was a qualitative enzyme-linked immunosorbent assay (ELISA) for the detection of total antibody to HBcAb in human serum or plasma. HBcAb appeared in virtually all individuals infected with HBV and was an accurate serological marker of current and past infection.

#### Hepatitis B surface antigen (HBsAg)

The AUSZYME Monoclonal test was a solid-phase “sandwich” enzyme immunoassay used to detect the presence of HBsAg, which indicated current infection with HBV.

#### Other measures

We considered gender, age, BMI, ethnic/race group, current smoker, current alcohol consumption, family history of diabetes, education, and poverty index as covariates. Age was recorded in years at the time of the screening interview. Gender was based on self-reported categories from the participants, as well as race/ethnicity which were categorized as Mexican Americans, other Hispanics, non-Hispanics whites, non-Hispanic blacks, and other ethnic/racial groups. BMI (kg/m^2^) was calculated from measured weight (kg) divided by the square of standing height (meter). Current smoker was defined as using tobacco/nicotine last 5 days or not. Current alcohol consumption was defined as at least 12 alcohol drinks per years or not in the past year. Family history of diabetes was defined as any of blood relatives including father, mother, sisters or brothers, ever told by a health professional that they had diabetes or not. Education was categorized based on the self-reported highest education as follows: less than 9th grade education, 9-11th grade education (includes 12th grade and no diploma), high school graduate, some college or associates degree, and college graduate or higher. Poverty index was an index for the ratio of family income to poverty based on the US Department of Health and Human Services’ poverty guidelines [13]. A poverty index of 1.0 represents the level of family income that is at the federal poverty level, and a poverty index of 2.0 represents a family income that is 200% of the federal poverty level.

### Statistical analysis

Continuous data were expressed in mean ± standard deviation. Continuous differences were examined using a two-tailed Student’s t-test. Categorical differences were given in proportions and examined using Chi-square test. Logistic regression analysis was used to calculate the odds ratio with 95% confidence intervals of diabetes based on vaccination history, HBV serology markers and successful HBV vaccination with the consideration of covariates as described above. In model 1, no covariates were considered; in model 2, gender, age, and BMI were considered; in model 3, ethnic/racial groups were entered in addition to gender, age, and BMI; in model 4, all covariates were entered. A nominal P value of < 0.05 was considered to be significant. SYSTAT 13.0 for windows package from SPSS, INC. (Chicago, IL, USA) was used for statistical analysis.

## Results

### Hepatitis B status

The clinical features of studied subjects were shown in Table 1. In this representative US population, the prevalence of HBV infection was 6.35% based on positive HBcAb, in contrast the prevalence of positive HBsAg was 0.40%. The prevalence of positive HBsAb was 19.66%. Among those with positive HBcAb, 6.28% were positive for HBsAg, while only 0.13% of HBsAb positive subjects were positive for HBsAg. Based on positive HBsAb and negative for both HBcAb and HBsAg, 2,320 subjects (15.15%) were immunized. As 4,063 subjects (26.53%) reported to receive HBV vaccination, successful HBV vaccination rate was 39.01% (Table 2).

**Table 1:** Clinical and demographic characteristics.

		n	mean (n) STD (%)
Gender	female	15,316	7,905 (51.61%)
Age	year	15,316	49 ± 18
BMI	kg/m^2^	15,316	28.99 ± 6.73
Current alcohol consumption	yes	15,316	10,028 (65.47%)
Current smoking	yes	15,316	3,685 (24.06%)
Family history of diabetes	yes	15,316	6,127 (40.00%)
Poverty index*		15,316	2.82 ± 1.81
Education**		15,316	10,859 (70.90%)
Ethnic/racial group		15,316	
Mexican American			2,876 (17.57%)
Other Hispanic			1,305 (7.97%)
Non-Hispanic white			7,484 (45.71%)
Non-Hispanic black			2,945 (17.99%)
Others			706 (4.31%)
Fasting plasma glucose	mmol/L	7,421	6.0 ± 2.0
2-hour plasma gluccose	mmol/L	5,394	6.8 ± 3.0
HbA1c			
NGSP	%	15,307	5.7 ± 1.0
IFCC	mmol/mol	15,307	38.8 ± 10.9

Mean ± standard deviation, or n (percent).

*In reference to the level of family income that is at the federal poverty level as 1.00.

**High school graduate or higher.

**Table 2:** Distribution of hepatitis B vaccination by serological evidence of immunization for hepatitis B.

		Hepatitis B vaccination					
		Yes		No		Subtotal	
Imminzation for hepatitis B	Yes	1,587	(39.06%)	733	(6.51%)	2,320	(15.15%)
Imminzation for hepatitis B	No	2,476	(60.94%)	10,520	(93.49%)	12,996	(84.85%)
	Subtotal	4,063	(26.53%)	11,253	(73.47%)	15,316	(100.00%)

Pearson Chi-Square = 2,460.1284, degree of freedom = 1.000, P<0.001

Hepatitis B vaccination: Yes, vaccination per self-report; No, no vaccination per self-report

Immunization for hepatitis B: Yes, positive for hepatitis B surface antibody and negative for both hepatitis B core antibody and surface antigen; No, others.

### Hepatitis B vaccination history and diabetes

Among 4,063 subjects with a history of HBV vaccination, diabetes was noted in 11.62%, in compared to 19.14% in those without a history of HBV vaccination (P<0.001, Table 3). The subjects with a history of vaccination were more female (P<0.001), younger (P<0.001), more current alcohol consumption (P = 0.001), more current smokers (P = 0.02), and higher education (P<0.001) with better glucose profiles (P<0.001) when compared to those without a history of HBV vaccination (Table 4). Analysis with covariates revealed age, gender, BMI, education, current alcohol consumption, and family history of diabetes had significant impacts (P<0.05) on the results while ethnic/racial groups, poverty index, and current smoking had not. However, the logistic regression analyses did not confirm its protective effect for diabetes (Table 3).

**Table 3:** Odds ratio and 95% confidence intervals for the risk of diabetes by vaccination and immunization of hepatitis B.

		Diabetes		Model 1	Model 2	Model 3	Model 4
		n	%	OR (95% CI)	OR(95% CI)	OR (95% CI)	OR (95% CI)
Vaccination	Yes	472	(11.62%)	0.56	1.01	1.03	1.08
Vaccination	No	2,154	(19.14%)	(0.50–0.62)	(0.89–1.14)	(0.91–1.16)	(0.96–1.23)
Immunization	Yes	156	(6.72%)	0.31	0.68	0.69	0.75
Immunization	No	2,470	(19.01%)	(0.26–0.36)	(0.56–0.95)	(0.58–0.83)	(0.62–0.90)
Successfu vaccination	Yes	85	(5.36%)	0.25	0.57	0.59	0.67
Successful vaccination	No	2,541	(18.51%)	(0.20–0.31)	(0.45–0.72)	(0.46–0.75)	(0.52–0.84)

Hepatitis B vaccination: Yes, vaccination per self-report; No, no vaccination per self-report

Hepatitis B immunization: Yes, positive for hepatitis B surface antibody and negative for both hepatitis B core antibody and surface antigen; No, others

OR, odds ratio; 95% CI, 95% confidence intervals

Model 1, unadjusted; Model 2, adjusted for gender, age, and BMI; Model 3, adjusted for gender, age, BMI, and ethnic/racial group; Model 4, adjusted for gender, age, BMI, ethnic/race group, active smoker, active alcohol consumption, family history of diabetes, poverty index, and education.

**Table 4:** Comparison of clinical features by history of hepatitis B vaccination.

		Hepatitis B vaccination		P
		Yes	No	
n		4,063	11,253	
Gender	female	2,392 (58.87%)	5,513 (48.99%)	<0.001
Age	year	40 ± 16	53 ± 18	<0.001
Body mass index	kg/m^2^	29.06 ± 7.14	28.96 ± 6.57	0.41
Current alcohol consumption	yes	2,722 (66.99%)	7,306 (64.92%)	0.001
Current smoker	yes	1,032 (25.40%)	2,653 (23.58%)	0.02
Family history of diabetes	yes	1,598 (39.33%)	4,529 (40.24%)	0.31
Poverty index*		2.80 ± 1.79	2.82 ± 1.81	0.52
Education**		3,218 (79.20%)	7,661 (68.08%)	<0.001
Ethnic/racial group				<0.001
Mexican American		671 (16.51%)	2,686 (19.56%)	
Other Hispanic		389 (9.58%)	1,189 (8.66%)	
Non-Hispanic white		1,826 (44.94%)	6,639 (48.36%)	
Non-Hispanic black		935 (23.01%)	2,620 (19.08%)	
Others		242 (5.96%)	595 (4.33%)	
Fasting plasma glucose***	mmol/L	5.7 ± 1.6	6.1 ± 2.1	<0.001
2-hour plasma glucose***	mmol/L	6.1 ± 2.4	7.0 ± 3.1	<0.001
HbA1c***				
NSPG	%	5.5 ± 0.9	5.8 ± 1.1	<0.001
IFCC	mmol/mol	36.6 ± 9.8	39.9 ± 12.0	<0.001

Mean ± standard deviation, or n (percent).

Hepatitis B vaccination: Yes, vaccination per self-report; No, no vaccination per self-report

*In reference to the level of family income that is at the federal poverty level as 1.00.

**High school graduate or higher.

***For fasting plasma glucose, n = 1,989 for those with hepatitis B vaccination and n = 5,432 for those without hepatitis B vaccination; for 2-hour plasma glucose, n = 1,476 and n = 3,918, respectively; for HbA1c, n = 4,062 and n = 11,245, respectively.

### Hepatitis B immunization and diabetes

In this population, 15.15% of the subjects showed serological evidence of HBV immunization. Among them, 6.72% were diabetic while diabetes was noted in 19.01% of the subjects without serological evidence of HBV immunization (P<0.001). Again, there were significant differences in the clinical features between the two groups (Table 5). Although the logistic regression analyses confirmed the influence of covariates and ORs increased, the protective effect of HBV immunization remained persistent after adjustment of all covariates with an OR of 0.75 (95%CI: 0.62–0.90, Table 3). The impact of each covariate on the results was highly similar to the analysis of HBV vaccination history and diabetes with minor differences in P values.

**Table 5:** Comparison of clinical features by states of hepatitis B immunization per hepatitis B serology.

		Hepatitis B immunization		P
		Yes	No	
n		2,320	12,996	
Gender	female	1,438(61.98%)	6,467(49.76%)	<0.001
Age	year	37±16	52±18	<0.001
Body mass index	kg/m^2^	27.60±6.19	29.24±6.79	<0.001
Current alcohol consumption	yes	1,572(67.76%)	8,456(65.07%)	0.01
Current smoker	yes	563(24.27%)	3,122(24.02%)	0.80
Family history of diabetes	yes	829(35.73%)	5,298(40.77%)	<0.001
Poverty index*		2.93±1.81	2.80±1.80	<0.001
Education**		1,937(83.49%)	8,943(68.81%)	<0.001
Ethnic/racial group				<0.001
Mexican American		329(14.18%)	2,547(19.60%)	
Other Hispanic		182(7.84%)	1,123(8.64%)	
Non-Hispanic white		1,134(48.88%)	6,350(48.86%)	
Non-Hispanic black		492(21.21%)	2,453(18.88%)	
Others		183(7.89%)	523(4.02%)	
Fasting plasma glucose***	mmol/L	5.5±1.4	6.1±2.1	<0.001
2-hour plasma glucose***	mmol/L	5.9±2.1	6.9±3.1	<0.001
HbA1c***				
NSPG	%	5.4±0.8	5.7±1.1	<0.001
IFCC	mmol/mol	35.5±8.7	38.8±12.0	<0.001

Mean ± standard deviation, or n (percent).

Hepatitis B immunization: Yes, positive for hepatitis B surface antibody and negative for both hepatitis B core antibody and surface antigen; No, others

*In reference to the level of family income that is at the federal poverty level as 1.00.

**High school graduate or higher.

***For fasting plasma glucose, n = 1,123 for those with immunization to hepatitis B and n = 6,298 for those without immunization to hepatitis B; for 2-hour plasma glucose, n = 885 and n = 4,509, respectively; for HbA1c, n = 2,319 and n = 12,988, respectively.

### Successful hepatitis B vaccination and diabetes

Among those who were tested positive for HBsAb and negative for both HBcAb and HBsAg with a history of HBV vaccination, 1,587 (10.36%) subjects were deemed successfully vaccinated for HBV. The prevalence of diabetes was significantly lower in subjects with successful HBV vaccination than those without it (5.36% vs. 18.51%, P<0.001, Table 3), suggesting the protective effect for diabetes by successful HBV vaccination. Again two groups differed significantly in clinical features except for current smoker (Table 6). The logistic regression analyses confirmed the protective effects for diabetes (Table 3). Successful HBV vaccination was associated with a reduced odds of diabetes (OR: 0.67, 95%CI: 0.52–0.84). Again age, gender, BMI, education, current alcohol consumption, and family history of diabetes had significant impacts (P<0.05) on the results while ethnic/racial groups, poverty index, and current smoking had not.

**Table 6:** Comparison of clinical features by states of successful vaccination confirmed by hepatitis B serology.

		Successful vaccination for hepatitis B		P
		Yes	No	
n		1,587	13,729	
Gender	female	1,038(65.41%)	6,867(50.02%)	<0.001
Age	year	36±14	51±18	<0.001
Body mass index	kg/m^2^	27.74±6.18	29.13±6.77	<0.001
Current alcohol consumption	yes	1,097(69.12%)	8,931(65.05%)	0.001
Current smoker	yes	359(22.62%)	3,326(24.23%)	0.16
Family history of diabetes	yes	565(35.60%)	5,562(40.51%)	<0.001
Poverty index*		3.02±1.76	2.79±1.81	<0.001
Education**		1,404(88.47%)	9,455(68.87%)	<0.001
Ethnic/racial group				<0.001
Mexican American		190(11.97%)	2,686(19.56%)	
Other Hispanic		116(7.31%)	1,189(8.66%)	
Non-Hispanic white		845(53.25%)	6,639(48.36%)	
Non-Hispanic black		325(20.48%)	2,620(19.08%)	
Others		111(6.99%)	595(4.33%)	
Fasting glucose***	mmol/L	5.4±1.0	6.1±2.1	<0.001
2h glucose***	mmol/L	5.8±1.9	6.9±3.1	<0.001
HbA1c***				
NGSP	%	5.3±0.6	5.7±1.1	<0.001
IFCC	mmol/mol	34.4±6.6	38.8±12.0	<0.001

Mean ± standard deviation, or n (percent).

Successful vaccination for hepatitis B: Yes, positive for hepatitis B surface antibody and negative for both hepatitis B core antibody and surface antigen with a history of HBV vaccination; No, others

*In reference to the level of family income that is at the federal poverty level as 1.00.

**High school graduate or higher.

***For fasting glucose, n = 802 for those with successful vaccination and n = 6,619 for those without successful vaccination; for 2-hour plasma glucose, n = 650 and n = 4,744, respectively; for HbA1c, n = 1,586 and n = 13,721, respectively.

## Discussion

To investigate the role of HBV in diabetes, we examined the association of HBV vaccination history and HBV serology with diabetes in a representative US population. Our results suggest that serological evidence of HBV immunization is associated with reduced odds of diabetes (33%), and successful HBV vaccination, defined by a history of hepatitis B vaccination with positive HBsAb, negative HBcAb, and negative HBsAg, was a protective factor for diabetes. Since only a very small subset of subjects in this population were chronic HBV carriers (positive HBsAg, 0.40%), we were not able to conduct a meaning analysis of the odds of diabetes in the group with chronic HBV infection.

In this study, we had no information on the age of HBV vaccination. As the titer of HBV declines with time, the high vaccination failure rate in this sample set could be a time related issue. However, we could not exclude other causes from the limited information available. As shown in Table 6, subjects who were successful vaccination for HBV were more female, younger, leaner by BMI, of better financial status by poverty index, and completed higher education. HBV vaccination could be a selective process for a lower diabetes risk. If the observed association is from a self-selection of health behavior, we should observe the association of HBV vaccination alone with diabetes, regardless of HBV serological status. However, no association of HBV vaccination with diabetes was found in this population after adjustment for covariates (Table 3). In contrast, the protective effect against diabetes was only observed with serological evidence of HBV immunization and successful HBV vaccination. Thus, the observed association is not a result of self-selective process of heath behavior.

Furthermore, logistic regression analysis provided the means to examine the differences in covariates between the subjects with and without successful HBV vaccination. It revealed that ethnic/racial groups, poverty index, and active smoker had no effect on the reduced odds of diabetes (P>0.05), while significant effects (P<0.001) were noted for gender, age, BMI, current alcohol consumption, family history of diabetes, and education. Although a much lower risk (OR: 0.25) for those with successful HBV vaccination was noted in Model 1 (unadjusted analysis), the ORs increased drastically after adjustment for differences in gender, age, and BMI (Model 2 in Table 3). Additional adjustment with other covariates only provided a fairly modest change in the OR. The results confirmed that after adjustment for covariates, successful HBV vaccination was independently associated with reduced odds of diabetes: 0.67 (95% CI: 0.52–0.84).

Among 2,626 diabetic subjects included in this study, 819 subjects were undiagnosed prior to taking the survey. As the concern of undiagnosed may represent a different group, we also examined the association based on 1,807 subjects with established diabetes only. The OR for HBV vaccination within the subjects with established diabetes was 1.12 (95% CI: 0.97–1.29), 0.74 (95% CI: 0.60–0.93) for those with HBV immunization by serological evidence, and 0.63 (95%CI: 0.47–0.84) for those with successful hepatitis B vaccination by both HBV vaccination history and serological evidence of immunization after adjustment for all covariates. The results were very consistent with the analysis based on all 2,626 diabetic subjects.

The major strength of the current study is derived from a fairly large representative US population. Furthermore, the results of HBV vaccination and successful HBV vaccination are complimentary to each other. The major limitation of the current study is that it is a retrospective association study. The causal relationship of diabetes and HBV is unknown as well as underlying molecular mechanisms. Furthermore, the low rate of successful immunization in diabetic subjects after HBV vaccination (16.77% vs. 41.02% in non-diabetic subjects), as noted in other studies [14,15], could also play some role in this association. Diabetes is associated with an increased risk for acute HBV infection [16]. Although the role of HBV infection in the pathogenesis of diabetes is unknown, the underlying mechanistic insights could stem from several lines of evidence. The liver plays a key role in maintaining glucose homeostasis, including insulin-mediated processes and the clearance of insulin itself [17]. Various liver diseases are associated with diabetes [18,19]. Furthermore, the association of hepatitis C virus infection and diabetes is well recognized [4]. Insulin resistance is associated with hepatitis C virus infection and has been inferred to play a role in the pathogenesis of diabetes [20]. The presence of hepatitis C virus in human pancreatic beta cells is associated with morphological cell changes and beta cell dysfunction [21]. Additionally, inflammation with the production of pro-inflammatory cytokines has also been demonstrated in diabetic patients with hepatitis C virus infection [22]. Thus, HBV infection might increase the risk of diabetes through similar mechanisms and, accordingly, immunization with HBV might reduce the risk of diabetes.

It has been well recognized that the primary purpose of vaccination is to reduce infectious disease and its sequelae. In addition to reduction of HBV infection, it has been demonstrated that vaccination with HBV also reduces the incidence of hepatocellular carcinoma [23]. This study further demonstrates an additional potential benefit of HBV vaccination by reducing the risk of diabetes, which is an unforeseen bonus of HBV vaccination, as if confirmed by an interventional study.

Diabetes can be delayed or prevented by various ways. The Diabetes Prevention Program demonstrates that lifestyle intervention reduces the incidence of diabetes by 58% and metformin by 31%, as compared to placebo after an average of 2.8 years [24]. The similar effect of behavior modification on the reduction of diabetes was also noted from other studies [25–27]. In addition to metformin, various pharmacological interventions are also very effective in the prevention of diabetes [28–30]. However, it requires significant cost and commitment to achieve the target goal of diabetes prevention [31]. Furthermore, cost-effectiveness of diabetes prevention through the above mentioned behavior modification has been challenged [32]. Once an intervention study is conducted and confirms the observation of our study, HBV vaccination may potentially act as a relatively cheap and cost-effective method of reducing the risk of diabetes in addition to the current recommendations of behavior and lifestyle modifications.

In this representative US population, the HBV vaccination rate was relatively low with only 26.53% of subjects having been vaccinated and almost three-fourths of subjects aged 20 years or older were not vaccinated for HBV. The first vaccine for HBV was approved by the FDA in 1981 and the CDC recommended HBV vaccination for all newborns in the United States in 1991. In this study, we included subjects 20 years or older in the NHANES from 2005 to 2010. Thus, it is very unlikely the subjects vaccinated in this population were the results of the national newborn vaccination program. With a relatively low HBV vaccination rate in the population aged 20 years or older, it provides a unique opportunity to intervene the epidemic of diabetes through HBV vaccination.

## Conclusion

The results of this study suggest that successful HBV vaccination (positive HBsAb, negative HBcAb, and negative HBsAg with HBV vaccination) is associated with reduced odds of diabetes by 33%. As the HBV vaccination rate is relatively low (26.53%) in the current US population aged 20 years or older, prevention of diabetes through HBV vaccination is a relatively low cost and can be easily achieved, as compared to known measures that require long-term commitment of behavior modification and/or costly pharmacological intervention. Furthermore, about 50% of projected increases in the prevalence of diabetes by 2030 will occur in developing countries, especially Asian and Pacific regions [3], where hepatitis B has become an epidemic [8]. Thus, HBV vaccination could have a significant impact on the epidemic of diabetes in these countries. An intervention trial is warranted before promoting a large scale application of HBV vaccination for prevention of diabetes.
